# Refractive error is associated with intracranial volume

**DOI:** 10.1038/s41598-017-18669-0

**Published:** 2018-01-09

**Authors:** Hikaru Takeuchi, Yasuyuki Taki, Rui Nouchi, Ryoichi Yokoyama, Yuka Kotozaki, Seishu Nakagawa, Atsushi Sekiguchi, Kunio Iizuka, Yuki Yamamoto, Sugiko Hanawa, Tsuyoshi Araki, Carlos Makoto Miyauchi, Takamitsu Shinada, Kohei Sakaki, Yuko Sassa, Takayuki Nozawa, Shigeyuki Ikeda, Susumu Yokota, Magistro Daniele, Ryuta Kawashima

**Affiliations:** 1 Division of Developmental Cognitive Neuroscience, Institute of Development, Aging and Cancer, Tohoku University, Sendai, Japan; 2 Division of Medical Neuroimaging Analysis, Department of Community Medical Supports, Tohoku Medical Megabank Organization, Tohoku University, Sendai, Japan; 3 Department of Radiology and Nuclear Medicine, Institute of Development, Aging and Cancer, Tohoku University, Sendai, Japan; 4 Creative Interdisciplinary Research Division, Frontier Research Institute for Interdisciplinary Science, Tohoku University, Sendai, Japan; 5 Human and Social Response Research Division, International Research Institute of Disaster Science, Tohoku University, Sendai, Japan; 6 Smart Ageing International Research Center, Institute of Development, Aging and Cancer, Tohoku University, Sendai, Japan; 7 School of Medicine, Kobe University, Kobe, Japan; 8 Division of Clinical research, Medical-Industry Translational Research Center, Fukushima Medical University School of Medicine, Fukushima, Japan; 9 Department of Functional Brain Science, Institute of Development, Aging and Cancer, Tohoku University, Sendai, Japan; 10 Division of Psychiatry, Tohoku Pharmaceutical University, Sendai, Japan; 11 Department of Adult Mental Health, National Institute of Mental Health, National Center of Neurology and Psychiatry, Tokyo, Japan; 12 Department of Psychiatry, Tohoku University Graduate School of Medicine, Sendai, Japan; 13 Graduate School of Arts and Sciences, Department of General Systems Studies, The University of Tokyo, Tokyo, Japan; 14 Department of Ubiquitous Sensing, Institute of Development, Aging and Cancer, Tohoku University, Sendai, Japan; 15 National Centre for Sport and Exercise Medicine (NCSEM), The NIHR Leicester-Loughborough Diet, Lifestyle and Physical Activity Biomedical Research Unit, School of Sport, Exercise, and Health Sciences, Loughborough University, Loughborough, England

## Abstract

Myopia is part of the spectrum of refractive error. Myopia is associated with psychometric intelligence and, the link between brain anatomy and myopia has been hypothesized. Here we aimed to identify the associations between brain structures and refractive error in developed young adults. In a study cohort of 1,319 normal educated young adults, the refractive error showed a significant negative correlation with total intracranial volume and total cerebrospinal fluid (CSF) volume but not with total gray matter volume (GMV) or total white matter volume (WMV). Time spent studying was associated with refractive error but could not explain the aforementioned associations with brain volume parameters. The R^2^ values of the simple regression between spherical equivalent and outcome variables for each sex in non-whole brain imaging analyses were less than 0.05 in all cases and thus were weak. Psychometric intelligence was not associated with refractive error or total CSF volume, but it weakly positively correlated with total GMV and total WMV in this study population. Thus, refractive error appears to be primarily (weakly) associated with the volume of the cranium, whereas psychometric intelligence was associated with the volume of the brain.

## Introduction

Myopia is the most common eye disorder and is part of the spectrum of refractive error, measured as the spherical equivalent. The spherical equivalent is the required quantitative strength of a spectacle lens to focus images on the retina of the eye^1^. A lower spherical equivalent reflects myopia. Myopia has become increasingly prevalent in modern life, especially in Asian countries; accordingly, this phenomenon as well as its cause has garnered a great deal of scientific attention^2^.

Myopia is associated with a number of important psychological variables. A previous study of people with substantially high IQs showed that 47% of females and 33% of males had very early onset myopia (i.e., by age 10) compared with approximately 5% of the control group with IQs in the normal range^3^. In addition, the association between myopia and psychometric intelligence was reported in numerous studies (for reviews, see refs^4–6^); however, a study with the large sample size (N > 1000) showed that this association was weak (r ≈ 0.1)^7^.

Along with psychometric intelligence, myopia is also associated with a narrow focus of visual attention^8^, and myopes experience a greater decrease in contrast sensitivity in the far periphery when attention is paid to central vision^9^. Perhaps due to less attentional resources in the peripheral field, myopia is also associated with defective automatic orienting of attention^10^. Associations between reduced visual acuity as well as refractive errors and personality traits, such as lower extraversion, were previously observed^11^^,^^12^. Finally, poorer visual acuity is associated with hallucinations^13^ and paranoid tendencies^14^. Thus, limited visual information may worsen these conditions.

It was previously suggested that the correlation between psychometric intelligence and myopia may be due to common genetic mechanisms affecting both brain size and eyeball size (as myopia’s proximate cause is a mismatch between the optical power of the eye and the axial length) or by the length of studying and reading required for high intelligence (^5^, for a recent review of the relevant hypothesis and relevant findings, see ref.^4^). The former speculation is partly based on the fact that the eye initially develops as an outgrowth of the brain^4,5^. Consistent with the genetic hypothesis, a twin study showed that the small correlation between refractive error and intelligence among 1500 adolescents (r = −0.116) was mainly (78%) explained by genetic factors^7^. With regards to the environmental mechanisms, human and animal studies showed that near-work, studying and reading and not seeing distant places, and less participation in sports are associated with myopia (for reviews, see refs^4^^,^^15^).

Previous neuroimaging studies investigated the regional gray/white matter density^16^ and regional gray matter volume (rGMV)^17^ of patients with myopia in a small sample population (N ≈ 60, including controls) without corrections for multiple comparisons across the whole brain^16^ or using a method that turned out to be incapable of correcting for multiple comparisons (Monte Carlo simulation using the AlphaSim program)^18^. Of these, one study comparing 30 highly myopic young adults and 30 control subjects reported a tendency toward an increased regional white matter density in the calcarine cortex of myopic subjects^16^, whereas another study comparing 27 highly myopic young adults and 32 control subjects reported an association between visual acuity and rGMV^17^. The emergence of large structural studies with small effects revealed that large sample sizes are required to detect associations between various individual parameters and brain structures in healthy subjects^19^. Thus, the association between myopia or refractive error and brain volume measurements, including total brain size, was not detected in previous studies.

We speculated on the potential associations between myopia and brain anatomy as follows. First, based on the abovementioned traditional hypotheses that the brain and eyeball have common genetic and developmental mechanisms, which explains the association between myopia and intelligence^4,5^, myopia may be associated with a greater total brain size. In addition, the defective automatic orienting of attention in subjects with myopia suggests that neural correlates of automatic attentional reorienting, such as the temporoparietal junction^20^, are associated with myopia. Also, myopia may be associated with hippocampal structures involved in studying^21^ and exercise^22^ which are positively and negatively associated with myopia, respectively. Finally, the brain structures of the basal ganglia, which is associated with personalities such as extraversion^23^, may be also associated with refractive error, too because extraversion is associated with refractive error as described above.

The purpose of this study was to and determine the associations between structural brain characteristics and refractive error. In addition, we investigated the associations between the neural correlates and psychological correlates (personality traits, daily habits, and other myopia-related traits) of myopia.

## Methods

### Subjects

The present study, which is a part of an ongoing project to investigate the association between brain imaging, cognitive function, and aging, refractive error and structural data from 1,319 healthy, right-handed individuals (763 men and 556 women). The mean age of the subjects was 20.8 years [standard deviation (SD), 1.8; age range: 18–27 years old]. For details of subjects’ information, see Supplemental Methods. Written informed consent was obtained. All methods were performed in accordance with the Declaration of Helsinki (1991). This study was approved by the Ethics Committee of Tohoku University.

### Measurement of the spherical equivalent

Refractive error was assessed by measuring the spherical equivalent. In this study, the refractive error of each study subjects was assessed using an auto refractometer (Shin-Nippon ACCUREF 8001 Auto Refractometer, Ajinomoto Trading Inc.; Tokyo, Japan).

### Psychological measures

Neuropsychological tests and questionnaires were administered. Details of these tests are described in previous studies, and the description in this subsection was mostly reproduced from our previous studies^24^. [A] Raven’s Advanced Progressive Matrices (RAPM)^25^ is a non-verbal reasoning task and a representative measure of general intelligence. [B] The Tanaka B-type intelligence test was performed as previously described^26^. This test calculates psychometric intelligence from several speeded tasks. [C] Reading comprehension task was developed by Kondo *et al*.^27^. For more details on this task, such as how it was developed and its validity, please refer to Kondo *et al*.^27^ and our previous study^24^. [D] The SA creativity test^28^ measures creativity via divergent thinking. For additional details, please refer to our previous study^29^. [E] The (computerized) digit span task is a working memory task. For details, see our previous study^30^. [F] *NEO Five-Factor Inventory* (NEO-FFI)^31^ is a questionnaire that was used to measure the five factors of personality. [G] The External-Preoccupation Scale^32^ is a questionnaire that was used to measure the maintenance of external focus on specific issues. [H] The Japanese version of the Paranoia Checklist is a questionnaire that was used to measure paranoid ideation. This questionnaire comprised 18 items, and each item contained 3 questions (frequency, conviction, and distress). These questions are answered using a 5-point Likert scale (1–5). The sum of these answers yields scores for frequency, conviction, and distress. For the reliability and validity of this scale, please see the previous study^33^. Higher subscores indicated stronger tendencies^33^. [I] The average time spent completing specific daily habits for 1 week during the previous month was obtained using a self-report questionnaire. We obtained data on time spent studying at home and the library because a previous study showed the association between time spent studying and myopia. We determined the time spent reading because viewing close objects, such as when reading, studying, and watching TV, was related to myopia^15^. [J] Subjects’ average daily physical activity level from the previous month was collected using a self-report questionnaire. For details, please see our previous study^34^.

### Behavioral data analysis

The behavioral data were analyzed using SPSS 22.0 statistical software (SPSS Inc., Chicago, IL). The descriptions in this subsection were mostly reproduced from our previous study^35^. Associations between the spherical equivalent and target variables were analyzed using multiple regression analyses with age and sex as covariates. In addition to the normal SPSS-based multiple regression analyses, p-values were assessed with permutation (5000 iterations)-based multiple regression analyses using the ImPerm package^36^ and R software, version 3.4.1^37^ due to the atypical distribution of the spherical equivalent as permutation tests do not require normal distribution^38^. These analyses were used to confirm whether significant findings held true with robust statistics.

In these analyses, results with a threshold of *P* < 0.05 were considered to be statistically significant, after correcting for the false discovery rate (FDR) using the graphically sharpened method^39^.

### Image acquisition

The methods for MR image acquisition were described in our previous studies and reproduced below^40,41^. All MRI data acquisition was performed using a 3-T Philips Achieva scanner. High-resolution T1-weighted structural images (T1WIs: 240 × 240 matrix, TR = 6.5 ms, TE = 3 ms, FOV = 24 cm, slices = 162, slice thickness = 1.0 mm) were collected using a magnetization-prepared rapid gradient echo sequence. Diffusion-weighted data were acquired using a spin-echo EPI sequence (TR = 10293ms, TE = 55 ms, FOV = 22.4 cm, 2 × 2 × 2 mm^3^ voxels, 60 slices, SENSE reduction factor = 2, number of acquisitions = 1). The diffusion weighting was isotropically distributed along 32 directions (*b* value = 1,000 s/mm^2^). Additionally, the diffusion weighting was isotropically distributed along 32 directions (b value = 1,000 s/mm2). Additionally, three images with no diffusion weighting (b value = 0 s/mm2) (b = 0 images) and one b = 0 image were acquired from 1197 and 122 subjects, respectively, (TR = 10293 ms, TE = 55 ms, FOV = 22.4 cm, 2 × 2 × 2 mm^3^ voxels, 60 slices). FA and MD maps were calculated from the collected images using a commercially available diffusion tensor analysis package on the MR console. For more details, see Supplemental Methods.

### Pre-processing of structural data

We used voxel-based morphometry (VBM) 20 to evaluate rGMV and rWMV. Preprocessing of the T1WIs data was performed using Statistical Parametric Mapping software (SPM12; Wellcome Department of Cognitive Neurology, London, UK) implemented in Matlab (Mathworks Inc., Natick, MA, USA). The methods for the preprocessing of T1WIs were described in our previous studies and reproduced below^42^. Using the new segmentation algorithm implemented in SPM12, T1-weighted structural images of each individual were segmented and normalized to the Montreal Neurological Institute (MNI) space to give images with 1.5 × 1.5 × 1.5 mm^3^ voxels using diffeomorphic anatomical registration through exponentiated lie algebra (DARTEL) registration process implemented in SPM12. In addition, we performed a volume change correction (modulation)^43^. Subsequently, generated rGMV and rWMV images were smoothed by convolving them with an isotropic Gaussian kernel of 8 mm full width at half maximum (FWHM). For full descriptions of these procedures, see Supplemental Methods.

Microstructural properties of the brain were assessed using the mean diffusivity (MD) and fractional anisotropy (FA) values obtained from diffusion tensor imaging (DTI). As previously summarized^41^, lower MD is associated with greater tissue density, which can be caused by the increased presence of unspecific cellular structures (i.e., capillaries, synapses, spines, and macromolecular proteins); the properties of myelin, neuronal membrane, and axons; the shape of neurons and/or glia; and enhanced tissue organization^44,45^. In contrast, FA is strongly associated with microstructural properties related to brain structural connectivity^44^. Preprocessing and analysis of diffusion data were performed using Statistical Parametric Mapping (SPM) 8 implemented in Matlab. The methods for the preprocessing of diffusion data were described in our previous study and reproduced below^41^. Basically, we normalized MD, FA, gray matter segment [regional gray matter density (rGMD) map], white matter segment [regional white matter density (rWMD) map], cerebrospinal fluid (CSF) segments [regional CSF density (rCSFD) map] of diffusion images of subjects with a previously validated, modified version of the diffeomorphic anatomical registration through exponentiated lie algebra (DARTEL)-based registration process^46^ method to give images with 1.5 × 1.5 × 1.5 mm^3^ voxel size, then normalized MD images were masked by the custom mask image that is highly likely to be the gray or white matter, and normalized FA images were masked by the custom mask image that is highly likely to be the white matter and smoothed [FA images were smoothed by Gaussian Kernel of 6-mm full width at half maximum (FWHM) and the rest of the images were smoothed by Gaussian Kernel of 8-mm full width at half maximum (FWHM)]. For more details of preprocessing, see Supplementary Methods.

### Whole-brain statistical analysis

Using VBM, we investigated if the rGMV was associated with individual differences in the average spherical equivalent of both eyes. The statistical analyses of imaging data were performed with SPM8. In these analyses, we performed whole brain multiple regression analyses. These analyses were performed with sex, age, and spherical equivalent as covariates. We included only voxels with a signal intensity greater than 0.05 for each participant to analyze the rGMV and rWMV.

Using DTI, we investigated the FA and MD associated with individual differences in the average spherical equivalent of both eyes. The statistical analyses of imaging data were performed with SPM8. In these analyses, we performed whole brain multiple regression analyses. These analyses were performed with sex, age, number of b = 0 images, and spherical equivalent as covariates. The analyses of MD were limited to the gray + white matter mask that was created above. The analyses of FA were limited to the white matter mask that was created above.

A multiple comparison correction was performed using threshold-free cluster enhancement (TFCE)^47^ with randomized (5,000 permutations) nonparametric testing using the TFCE toolbox (http://dbm.neuro.uni-jena.de/tfce/). We applied a threshold of FWE corrected at *P* < 0.05.

## Results

### Basic data

**Figure 1:**
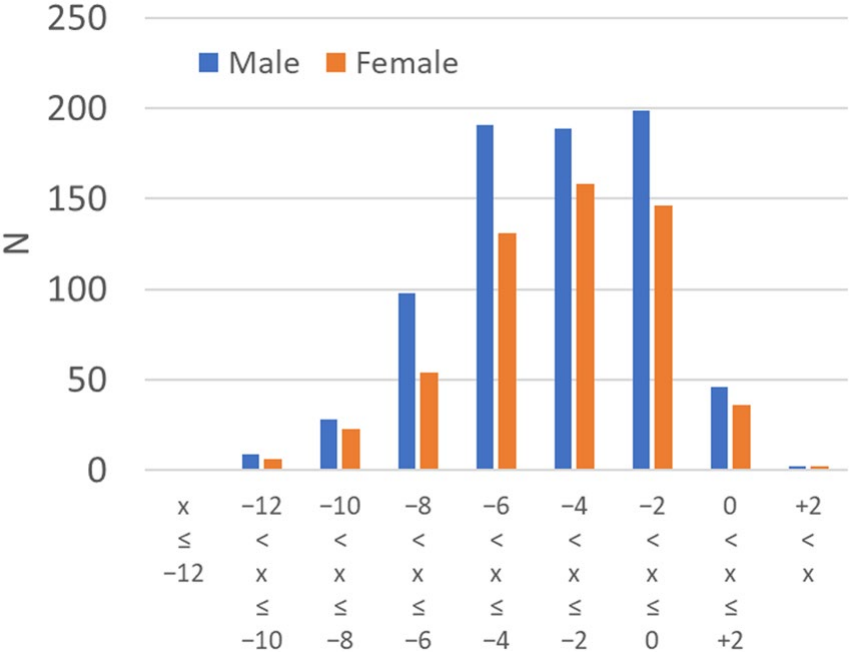
Distribution of spherical equivalent (D) in men and women.

The mean and standard deviation for age, general intelligence score, total GMV, total WMV, total CSF volume, total intracranial volume (TIV), and spherical equivalent within each sex are presented in Table 1. The spherical equivalent distribution in men and women are presented in Fig. 1.

**Table 1:** Demographics of study participants.

Measure	Male (n = 763)		Female (n = 556)	
	Mean	SD	Mean	SD
Age	20.85	1.88	20.70	1.62
RAPM	28.71	3.84	28.02	3.82
Spherical equivalent (D)	−3.51	2.62	−3.35	2.57
Total GMV	752.72	53.20	685.10	45.73
Total WMV	457.03	38.75	411.05	32.50
Total CSFV	406.23	73.21	335.86	63.15
TIV	1,615.98	117.86	1,432.01	92.98

### Psychological analyses of the correlation of the spherical equivalent with total brain volume measurements and psychological variables

**Figure 2:**
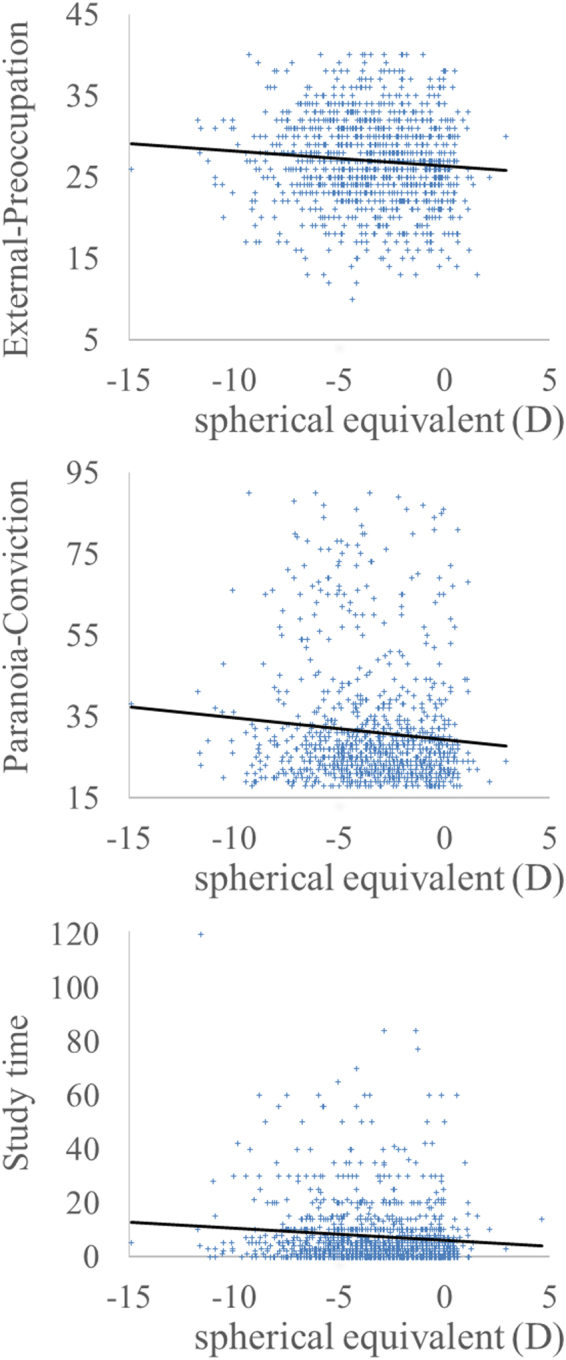
Scatter plots of associations between the spherical equivalent and psychological measurements. (**a**) The spherical equivalent showed a significant negative correlation with external preoccupation. (**b**) The spherical equivalent showed a significant negative correlation with conviction level of paranoid ideation. (**c**) The spherical equivalent showed a significant negative correlation with study time.

**Figure 3:**
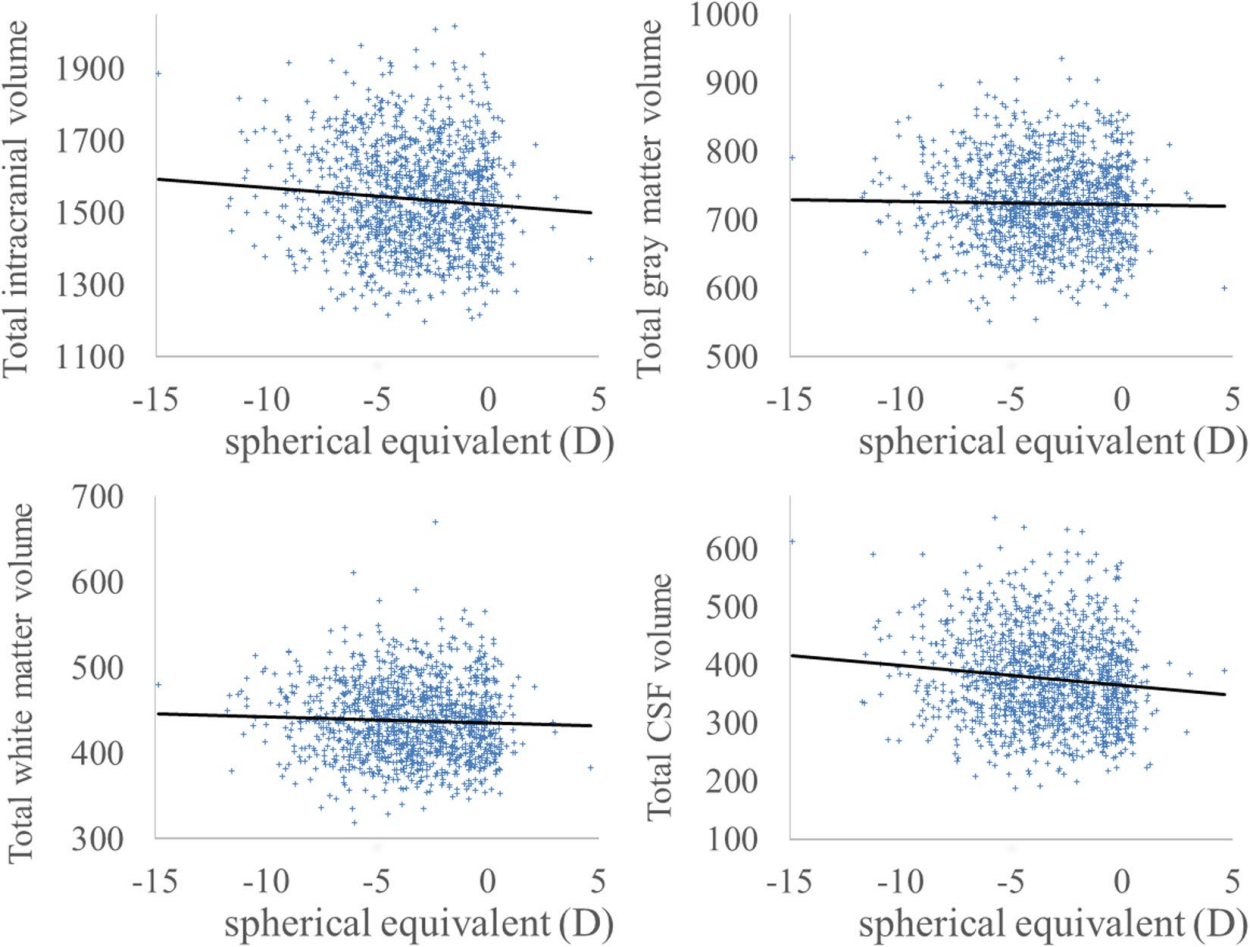
Scatter plots of associations between spherical equivalent and total brain volume measurements. (**a**) The spherical equivalent showed a significant negative correlation with total intracranial volume (**b**) The spherical equivalent did not significantly correlate with total GMV. (**c**) The spherical equivalent did not significantly correlate with total WMV. (**d**) The spherical equivalent showed a significant negative correlation with total CSF volume.

When the ordinary SPSS-based multiple regression analyses were used, after correcting for age and sex, the spherical equivalent showed significant negative correlations with TIV, total CSF volume, External-Preoccupation score, and weekly amount of time spent studying at home and in the library and significant positive correlations with the conviction score of the Paranoia Checklist (P < 0.05, corrected for multiple comparisons using false discovery rate, Figs 2 and 3). The spherical equivalent did not correlate with total GMV, total WMV, cognitive function including two intelligence tests, the five major factors of personality, or the weekly amount of time spent reading and watching TV.

Due to the atypical distribution of the spherical equivalent, the results were also confirmed using robust statistics. When permutation-based multiple regression analyses were used, all corrected results (from the ordinary multiple regression analyses) remained significant. In addition, the spherical equivalent showed a significant positive correlation with daily exercise habits (P < 0.05, corrected for multiple comparisons using false discovery rate). Although, significant findings with ordinary statistical tests were regarded as the formal result, these additional tests were congruent with previously reported negative associations between myopes and participation in sports^4^.

For statistical values, see Table 2.

**Table 2:** Statistical results of simple regression analyses in each sex (R^2^) and (R^2^, beta value, *t*-value, uncorrected *p*-values, *p*-value corrected for FDR^a^ of normal multiple regression analyses and permutation-based multiple regression analyses) for the multiple regression analyses performed using the psychological variables and the covariates of age, sex, and SE as dependent variables.

Dependent variables	N	SE		SE						
		Simple regression		Multiple regression						
		R^2^ male	R^2^ female	Adjusted R^2^	β	*t*	*p* (*normal*, *uncorrected*)	*p (normal, FDR)*	*p (Permutation, uncorrected)*	p (Permutation, FDR*)
Total intracranial volume	1319	0.00991	0.00477	0.419	−0.066	−3.114	0.002	0.012	<2 × 10^−16^	8.5 × 10^−4^
Total gray matter volume	1319	6.17 × 10^−4^	7.86 × 10^−4^	0.317	−0.009	−0.413	0.680	0.612	0.843	0.773
Total white matter volume	1319	1.64 × 10^−4^	0.00446	0.286	−0.024	−1.038	0.299	0.427	1	0.773
Total CSF^b^ volume	1319	0.0184	0.00768	0.224	−0.099	−4.063	5.12 × 10^−5^	9.21 × 10^−4^	<2 × 10^−16^	8.5 × 10^−4^
RAPM^c^	1319	1.48 × 10^−4^	3.44 × 10^−4^	0.006	−0.002	−0.075	0.940	0.769	1	0.773
TBIT^d^	1147	5.10 × 10^−5^	0.00291	0.028	−0.015	−0.510	0.610	0.610	1	0.773
Reading comprehension	1023	7.32 × 10^−7^	0.00206	9.87 × 10^−4^	−0.021	−0.656	0.512	0.542	0.196	0.278
S-A creativity test	1319	0.00296	0.00364	0.010	0.011	0.417	0.677	0.612	0.491	0.591
Digit span	1260	0.00312	0.00183	0.021	0.027	0.971	0.332	0.427	1	0.773
NEOFFI - neuroticism	1206	0.00102	1.66 × 10^−7^	0.028	−0.028	−0.994	0.320	0.427	0.099	0.210
NEOFFI - extraversion	1206	0.00319	0.00214	0.013	0.021	0.746	0.456	0.513	0.283	0.370
NEOFFI-openness	1206	0.00280	0.00059	0.002	0.043	1.473	0.141	0.270	0.022	0.053
NEOFFI - agreeableness	1206	0.00502	2.12 × 10^−5^	0.051	0.047	1.673	0.094	0.242	0.116	0.219
NEOFFI - conscientiousness	1206	1.50 × 10^−4^	0.00100	0.009	−0.029	−1.025	0.305	0.427	0.541	0.591
External - Preoccupation	1111	7.41 × 10^−4^	6.08 × 10^−4^	0.010	−0.085	−2.827	0.005	0.018	0.006	0.017
Paranoia Checklist - Frequency	976	0.00105	0.00106	0.018	−0.029	−0.899	0.369	0.443	0.151	0.257
Paranoia Checklist - Conviction	976	0.0133	0.0411	0.008	−0.092	−2.886	0.004	0.018	<2 × 10^−16^	8.5 × 10^−4^
Paranoia Checklist - Distress	976	0.00787	3.50 × 10^−5^	0.004	−0.047	−1.482	0.139	0.270	0.191	0.278
Habit of TV viewing	1187	3.35 × 10^−4^	0.00101	0.016	−0.007	−0.228	0.820	0.703	0.556	0.591
Habit of studying at home and library	1195	0.00691	0.0162	0.041	−0.092	−3.230	0.001	0.009	<2 × 10^−16^	8.5 × 10^−4^
Habit of reading	1200	6.88 × 10^−5^	0.00978	0.006	0.042	1.442	0.150	0.270	0.660	0.660
Monthly exercise	1021	0.00332	0.00626	0.009	0.065	2.068	0.039	0.117	0.004	0.014

^a^False discovery rate. ^b^Cerebrospinal fluid. ^c^Raven’s advanced progressive matrices (a general intelligence task). ^d^Tanaka B-type intelligence test.

*For the calculation of FDR-adjusted p-values, uncorrected p-values < 2 × 10^−16^ were treated as 0.0002 (1/5000, once in 5000 iterations).

### Whole-brain analyses of the correlations between the spherical equivalent and regional neuroimaging measurements

**Figure 4:**
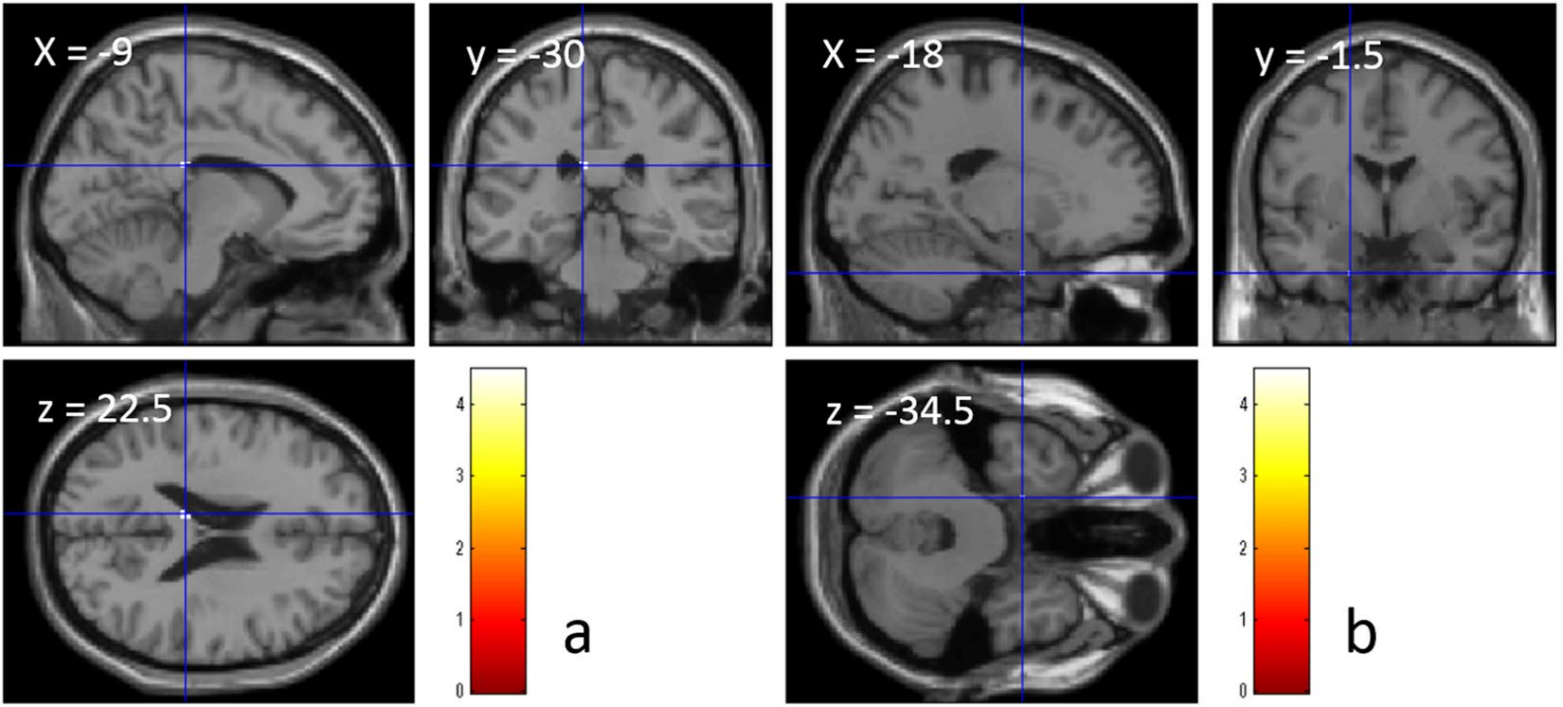
Brain regions with significant negative associations between mean diffusivity and spherical equivalent. (**a** and **b**) Results were obtained using a threshold of P < 0.05 corrected for multiple comparisons (voxel wise FWE). Results were corrected at the whole-brain level. Regions with significant positive correlations are overlaid on a “single subject” T1 image from SPM8. (**a**) Significant negative correlations with MD were found in the splenium of the corpus callosum. (**b**) Significant negative correlations with MD were found in the left parahippocampal gyrus.

A whole-brain multiple regression analysis correcting for confounding variables showed that the spherical equivalent did not significantly correlate with rGMV, regional white matter volume (rWMV), or FA. However, the spherical equivalent showed a significant negative correlation with MD in the splenium of the corpus callosum, left parahippocampal gyrus, and left temporal pole (Table 3, Fig. 4).

**Table 3:** Brain regions that showed a significant negative correlation between SE and MD.

	x	y	z	T value	Corrected *p* value (FWE)	Cluster size (voxels)
Corpus callosum of the splenium	−9	−30	22.5	4.49	0.029	14
Left parahippocampal gyrus	−18	−1.5	−34.5	4.44	0.035	1
Left temporal pole	−19.5	0	−36	4.38	0.044	1

### Correlations among the spherical equivalent, total volume measurements, clusters from whole brain analyses, and significant psychological correlates of spherical equivalent

We evaluated the associations among the spherical equivalent, total volume measurements, significant clusters from whole brain analyses, and significant psychological correlates of the spherical equivalent. MD in the left parahippocampal gyrus and splenium of the corpus callosum did not significantly correlate with the External-Preoccupation score, weekly amount of time spent studying at home and in the library, or Paranoia Checklist conviction score. No other correlations were observed between the remaining total volume measurements and psychological variables. See Table 4 for statistical values. Similar significant results were also confirmed using robust statistics (Statistical results were presented in Supplemental Table 1).

**Table 4:** Correlation coefficients and p-values for the associations between the spherical equivalent, significant correlated parameters, and total volume measurements.

	1	2	3	4	5	6	7	8	9	10
1 spherical equivalent	—									
2 total intracranial volume	−0.066, 0.002	—								
3 total gray matter volume	−0.009, 0.680	0.743, 8.08 × 10^−185^	—							
4 total white matter volume	−0.024, 0.299	0.824, 3.78 × 10^−234^	0.702, 5.60 × 10^−184^	—						
5 total CSF volume	−0.099, 5.12 × 10^−5^	0.791, 3.64 × 10^−181^	−0.006, 0.839	0.156, 5.73 × 10^−8^	—					
6 MD in the splenium	−0.123, 7.34 × 10^−6^	0.181, 4.44 × 10^−7^	0.042, 0.209	0.051, 0.116	0.191, 5.71 × 10^−×10^	—				
7 MD in the left temporal area	−0.094, 0.001	−0.078, 0.031	−0.101, 0.002	−0.107, 0.001	0.017, 0.596	0.079, 0.004	—			
8 preoccupation	−0.085, 0.005	−0.052, 0.181	−0.066, 0.069	−0.032, 0.365	−0.011, 0.742	0.049, 0.106	−0.031 0.301	—		
9 Paranoia Checklist - Conviction	−0.092, 0.004	0.047, 0.259	−0.004, 0.913	−0.016, 0.676	0.073, 0.043	0.029, 0.374	0.005, 0.869	0.005, 0.883	—	
10 study time	−0.092, 0.001	−0.081, 0.935	−0.036, 0.288	−0.019. 0.579	0.030, 0.351	0.014, 0.618	0.035, 0.214	0.037, 0.213	0.009, 0.770	—

In addition, we determined the associations between psychometric intelligence (RAPM and TBIT) and total volume measurements after correcting for age and sex. The RAPM score was unrelated to total GMV, total WMV, total CSF volume, and TIV in our sample population (P > 0.05). The TBIT score significantly correlated with total GMV (P = 0.004, β = 0.071, t = 2.896), total WMV (P = 0.001, β = 0.088, t = 3.480), and TIV (P = 0.003, β = 0.067, t = 2.952) but not total CSF volume (P = 0.479, β = 0.019, t = 0.708). Thus, psychometric intelligence and the spherical equivalent showed distinct relationships with total volume measurements.

## Discussion

We investigated the associations among structural brain measurements, cognitive differences, daily habits, and refractive error. Partly consistent with our hypothesis, we showed that a lower spherical equivalent was associated with a greater TIV. The spherical equivalent was associated with CSF volume but not with total or regional gray/white matter volume. In addition, a greater spherical equivalent was associated with lower MD in the splenium of the corpus callosum and left parahippocampal gyrus. The association between spherical equivalent and psychological intelligence was not observed in our sample population; however, a lower spherical equivalent was associated with the tendency to be preoccupied with external issues, increased paranoid conviction, and more time spent studying. Notably, the relationship between refractive error and these psychological measurements cannot explain the association of TIV and MD with refractive error. Notably, all correlations were weak, and R^2^ values of the simple regression between the spherical equivalent and outcome variables for each sex in non-whole brain imaging analyses were less than 0.05 (Table 2). Thus, the spherical equivalent explains only a minor part of each variant.

Our results suggest that a larger TIV, rather than a neural mechanism, is primarily associated with myopia. In the present study, the TIV showed a significant small negative correlation with the spherical equivalent. Among the parameters related to TIV, only the total CSF volume showed a significant but small negative correlation with the spherical equivalent. The total GMV and WMV did not correlate with the spherical equivalent. In contrast, psychometric intelligence (TBIT score) showed a significant positive correlation with TIV, total GMV, and total WMV but not total CSF volume. These results suggest that a larger brain is important for intelligence, whereas a larger cranium is weakly related to myopia. The exact mechanism of this phenomenon is not clear and outside the scope of our present study. In contrast to the previously hypothesized genetic and developmental mechanisms that could affect eyeball and brain because embryologically the eye is an outgrowth of the brain, our present results suggest that head size directly affects and is associated with the shape of the eyeball (spherical equivalent refraction is well-known to closely correlate with eye size measured along the optical axis and measuring spherical equivalent corresponds quite closely to a measure of eye size measured along the optical axis^4^). Future developmental studies should validate this speculation and elucidate the specific mechanisms involved. As described above, the previously reported small correlation between refractive error and psychometric intelligence is largely explained by genetic factors^7^ despite the evidence that environmental factors can also effect refractive error and intelligence^4^. This finding may be explained by the fact that head size can directly limit the size and shape of the eyes (physically). Alternatively, the cranium develops from the mesenchyme surrounding the neural tissue of the developing brain, and the scleral coat of the eye develops from the mesenchyme around the neural tissue of the retina^48^. Thus, the common developmental mechanism of these processes forms the correlation between spherical equivalent and cranial volume. An interesting congruence is that the intraocular volume of a myopic eye is greater than normal without a change in neural tissue (retinal) volume^4,49^; however, there is an increase in aqueous and vitreous volume, which parallels the association of spherical equivalent with intracranial volume and CSF volume but not brain volume (rGMV and rWMV). Yet another possibility is that the weak association between greater CSF and lower spherical equivalent could be due to hidden neurological problems that increase CSF, such as hydrocephalus. However, these neurological problems are also typically associated with reduced brain parenchyma, and an increase in CSF is not expected to result in an increased TIV. Thus, this hypothesis is not congruent with the results of our study.

We did not detect a significant association between the spherical equivalent and psychometric intelligence measurements. This finding is in conflict with that of some previous studies that showed the association between psychometric intelligence but is consistent with that of a previous study that showed the association between myopia and intelligence^4^; however, our results are consistent with studies that failed to show an association between non-verbal intelligence measurements and myopia^50^. Based on previous studies, it was suggested the associations between myopia and psychometric intelligence measures are particularly strongly seen when the verbal measures are used^50^. Our findings of the present lack of associations between the spherical equivalent and psychometric intelligence may be because we did not use assessments such as the Wechsler IQ test, which contains many verbal tasks^51^. Also, a recent review suggested the there was more evidence of the association of myopes and higher intelligence in younger children, (though the associations have also been confirmed in studies of huge sample size of young adults)^4^, which may explain the null findings in the present study. Alternatively, we found a weak association between spherical equivalent and study time in our highly educated sample population; however, substantial studying in the population may have weakened the association between psychometric intelligence and study time. We previously showed an association between verbal intelligence and study time in children from the general population^52^. In the present group of participants, we did not find significant associations between study time and psychometric intelligence measurements (RAPM and TBIT, P > 0.3 after correcting for age and sex). Future studies of the general population using verbal intelligence measurements may better reveal the associations among psychometric intelligence, brain volume, study time, and myopia. We also failed to observe an association between reading time and spherical equivalent. This finding is consistent with a portion of previous studies [for summary, see^53^]. The data on reading habits (hours/week) had a range of 0–40, and this range was apparently sufficient. This lack of association may be due to our use of a highly educated sample population. In our present results, the average time spent studying was much higher than the average time spent reading (7.50 h/week vs. 2.11 h/week), which may explain the weak effect of reading on the spherical equivalent.

The present study suggests another possibility for the greater prevalence of myopia in certain countries. The incidence of myopia differs across countries and cultures^4,50^. Notably, east Asians have a high incidence of myopia. These differences may be partly due to educational factors. However, education cannot explain all of the observed cultural differences related to myopia^54^. In addition, in this study, too, although, longer time spent studying is weakly associated with smaller spherical equivalent, time spent studying is not associated with total intracranial volume, nor total CSF volume, which were weakly associated with spherical equivalent. The incidence rates of myopia may be explained by differences in brain volume; indeed, east Asians have a relatively greater brain volume^55^.

The weak association between lower MD and a greater spherical equivalent in parahippocampal regions and the splenium of the corpus callosum may be due to use-dependent plasticity via increased visual information processing in subjects with a greater spherical equivalent. The splenium of the corpus callosum connects the bilateral parts of the posterior brain areas, including the visual cortex^56^. In addition, the parahippocampal gyrus is associated with the recognition of visual objects from the context of the surrounding scene^57^. Thus, increased visual information processing due to a greater spherical equivalent may increase the tissue of the splenium of the corpus callosum via use-dependent neural plasticity^45^ and lower MD. These speculations should be addressed in future studies.

The tendency of preoccupation toward external events was weakly associated with a lower spherical equivalent. This finding is consistent with previous studies showing that myopia is associated with a narrow focus of visual attention^8^ and defective automatic orienting of attention^10^. However, this preoccupation with external events was not significantly associated with the neural correlates of the spherical equivalent. The weak association between myopia and attentional processes may be mediated by different neural mechanisms, such as functional activity. Alternatively, the mechanisms behind myopia may lead to less distractive attentional mechanisms, similar to blinkers (a cup-shaped device to limit a horse’s vision and thus prevent it from swerving toward objects or other horses while racing) and may not be related to long term neural plasticity.

Indeed, in our study, the spherical equivalent (mean of both eyes) was measured with non-cycloplegic autorefraction (measured by a closed-field autorefractor) as was the case in some recent representative or relevant studies involving refractive error^1,7,58^. The procedure of cycloplegics was important for children, and to the best of our knowledge, a substantial portion of studies using adult samples has been conducted without this procedure. As was demonstrated by a previous study^59^ using a huge sample size, the difference in the refractive result for the spherical equivalent (measured by a non-open field autorefractor) with or without cycloplegia was around 0.5 D in adults. Thus, an additional cycloplegic examination would not have revealed major differences in our cohort. Further, we used regression analyses with continuous variables of the spherical equivalent; therefore, among the differences in spherical equivalent caused by this procedure, systematic bias (a difference introduced equally in all subjects) would not affect the results.

There are a few limitations of this study. First, the study participants were young and highly educated, and the majority of the sample population consisted of undergraduate and postgraduate students. This type of limited sampling is a common hazard of studies using college students^60^. As discussed in the Discussion section, this characteristic and the use of psychometric intelligence measures that are independent of verbal skills and knowledge may explain why our study did not observe the previously reported association between myopia and psychometric intelligence. However, this kind of focus on highly educated sample may make it possible to dissociate the effects of education level and those of study time; in this type of sample, psychometric intelligence and refractive error are not related, despite the association between total intracranial volume and refractive error. Therefore, whether the present findings can be applied to the general population needs to be confirmed in future studies.

Similar to the majority of relevant previous studies, we did not gather information on vision correction (e.g., when subjects began to correct their eyesight). This information was not included in the analysis, as was the case of almost all relevant studies. However, these data may aid in the interpretation of our findings.

In conclusion, a lower spherical equivalent, which reflects myopia, was weakly associated with greater TIV and total CSF volume. Study time was weakly related to lower spherical equivalent, but cannot explain the former association. In the present group of educated young adults, psychometric intelligence was not associated with refractive error or total CSF volume but did weakly positively correlate with total GMV and total WMV. Thus, refractive error appears to be primarily associated with the volume of cranium (albeit weakly), whereas psychometric intelligence is associated with the volume of the brain.

## Electronic supplementary material

Supplementary online material
