# Supplementary material for: Refractive error is associated with intracranial volume
Source: Sci Rep. 2018 Jan 9;8:175. doi: 10.1038/s41598-017-18669-0 (PMC5760524; doi:10.1038/s41598-017-18669-0)
Supplement: Supplementary file 1 — Supplementary online material [file 41598_2017_18669_MOESM1_ESM.pdf]

Refractive error is associated with intracranial volume

Hikaru Takeuchi[a], Yasuyuki Taki[a,b,c], Rui Nouchi[d,e,f], Ryoichi Yokoyama[g], Yuka Kotozaki[h], Seishu Nakagawa[i,j], Atsushi Sekiguchi[b,i,k], Kunio Iizuka[l], Yuki Yamamoto[i], Sugiko Hanawa[i], Tsuyoshi Araki[f], Carlos Makoto Miyauchi[m], Takamitsu Shinada[i], Kohei Sakaki[i], Yuko Sassa[a], Takayuki Nozawa[n], Shigeyuki Ikeda[n], Susumu Yokota[a], Magistro Daniele[o], Ryuta Kawashima[a,f,i]

[a]*Division of Developmental Cognitive Neuroscience, Institute of Development, Aging and Cancer, Tohoku University, Sendai, Japan*

[b]*Division of Medical Neuroimaging Analysis, Department of Community Medical Supports, Tohoku Medical Megabank Organization, Tohoku University, Sendai, Japan*

[c]*Department of Radiology and Nuclear Medicine, Institute of Development, Aging and Cancer, Tohoku University, Sendai, Japan*

[d]*Creative Interdisciplinary Research Division, Frontier Research Institute for Interdisciplinary Science, Tohoku University, Sendai, Japan*

[e]*Human and Social Response Research Division, International Research Institute of Disaster Science, Tohoku University, Sendai, Japan*

[f]*Smart Ageing International Research Center, Institute of Development, Aging and Cancer, Tohoku University, Sendai, Japan*

[g] *School of Medicine, Kobe University, Kobe, Japan*

[h]*Division of Clinical research, Medical-Industry Translational Research Center, Fukushima Medical University School of Medicine, Fukushima, Japan*

[i]*Department of Functional Brain Science, Institute of Development, Aging and Cancer, Tohoku University, Sendai, Japan*

[j] *Division of Psychiatry, Tohoku Pharmaceutical University, Sendai, Japan*

[k]*Department of Adult Mental Health, National Institute of Mental Health, National Center of Neurology and Psychiatry, Tokyo, Japan*

[l]*Department of Psychiatry, Tohoku University Graduate School of Medicine, Sendai, Japan*

[m]*Graduate School of Arts and Sciences, Department of General Systems Studies, The University of Tokyo, Tokyo, Japan*

[n]*Department of Ubiquitous Sensing, Institute of Development, Aging and Cancer, Tohoku University, Sendai, Japan*

[o] *National Centre for Sport and Exercise Medicine (NCSEM), The NIHR Leicester-Loughborough Diet, Lifestyle and Physical Activity Biomedical Research Unit, School of Sport, Exercise, and Health Sciences, Loughborough University, England*

Corresponding author:

Hikaru Takeuchi

Division of Developmental Cognitive Neuroscience, IDAC, Tohoku University

4-1 Seiryo-cho, Aoba-ku, Sendai 980-8575, Japan

Tel/Fax: +81-22-717-7988

E-mail: takehi@idac.tohoku.ac.jp

Key words:

myopia; spherical equivalent; brain volume; mean diffusivity; study time

Running title: Eyesight and brain anatomy

Supplemental online material

Supplemental Methods

Subjects. The present study, which is a part of an ongoing project to investigate the association between brain imaging, cognitive function, and aging, included spherical equivalent and structural data from 1,319 healthy, right-handed individuals (763 men and 556 women). The mean age of the subjects was 20.8 years [standard deviation (SD), 1.8; age range: 18-27 years old]. The following descriptions were mostly reproduced from another study of ours from the same project using the exactly same methods regarding these issues [1]. Some of the subjects who took part in this study also became subjects of our intervention studies (psychological data and imaging data recorded before the intervention were used in this study)[2]. Psychological tests and MRI scans not described in this study were performed together with those described in this study. All subjects were university students, postgraduates, or university graduates of less than one year's standing. All subjects had normal vision and none had a history of neurological or psychiatric illness. Handedness was evaluated using the Edinburgh Handedness Inventory [3]. Written informed consent was obtained from each subject. For nonadult subjects, written informed consent was obtained from their parents (guardians). This study was approved by the Ethics Committee of Tohoku University.

Subjects were instructed to get sufficient sleep, maintain their conditions, eat sufficient breakfast, and to consume their normal amounts of caffeinated foods and drinks in the day of cognitive tests and MRI scans. In addition, subjects were instructed to avoid alcohol the night before the assessment.

Pre-processing of structural data. The methods for the preprocessing of T1WIs were described in our previous study and reproduced below [4]. Preprocessing of the structural data was performed using Statistical Parametric Mapping software (SPM12; Wellcome Department of Cognitive Neurology, London, UK) implemented in Matlab (Mathworks Inc., Natick, MA, USA). Using the new segmentation algorithm implemented in SPM12, T1-weighted structural images of each individual were segmented into 6 tissues. In this new segmentation process, default parameters were used, except that the Thorough Clean option was used to eliminate any odd voxel, affine regularization was performed with the International Consortium for Brain Mapping template for East Asian brains, and the sampling distance was set at 1 mm. We then proceeded to the diffeomorphic anatomical registration through exponentiated lie algebra (DARTEL) registration process implemented in SPM12. We used DARTEL import images of the 2 TPMs from the abovementioned new segmentation process. First, the template for the DARTEL procedures was created using imaging data from 800 participants (400 males and 400 females). The following methods were the same as in our previous study and descriptions were reproduced from our previous study [5]. Next, using this existing template, the DARTEL procedures were performed for all of the subjects in the present study. In these procedures, default parameter settings were used. The resulting images were spatially normalized to the Montreal Neurological Institute (MNI) space to give images with $1.5 \times 1.5 \times 1.5$ mm^3 voxels. In addition, we performed a volume change correction (modulation) by modulating each voxel with the Jacobian determinants derived from spatial normalization, which allowed us to determine regional differences in the absolute amount of brain tissue [6]. Subsequently, normalized rGMV, rWMV, and rCSF volume images were smoothed by convolving them with an isotropic Gaussian

kernel of 8 mm full width at half maximum (FWHM).

Pre-processing of diffusion data

The methods for the preprocessing of diffusion data were described in our previous study and reproduced below [7]. Preprocessing and analysis of diffusion data were performed using Statistical Parametric Mapping (SPM) 8 implemented in Matlab. Using a previously validated two-step new segmentation algorithm of diffusion images and the previously validated diffeomorphic anatomical registration through exponentiated lie algebra (DARTEL)-based registration process [8], all images, including gray matter segment [regional gray matter density (rGMD) map], white matter segment [regional white matter density (rWMD) map], cerebrospinal fluid (CSF) segments [regional CSF density (rCSFD) map] of diffusion images, were normalized. The voxel size of normalized FA images and MD images and segmented images, was 1.5 × 1.5 × 1.5 mm^3. See our previous work [9] for the details of these procedures including the information of the template.

Next, we created average images of normalized rGMD and rWMD images from the normalized rGMD and rWMD images from the subset of the entire sample (63 subjects) [8]. Subsequently, for the analyses of MD images from the normalized images of the (a) MD, (b) rGMD, and (c) rCSFD maps, we created images where areas that were not strongly likely to be gray or white matter in our averaged normalized rGMD and rWMD images (defined by "gray matter tissue probability + white matter tissue probability < 0.99") were removed (to exclude the strong effects of CSF on MD throughout analyses). These images were then smoothed (8 mm full-width half-maximum) and carried through to the second-level analyses of MD.

Next, from the average image of normalized WM segmentation images from the 63 subjects mentioned above and from the created mask image consisting of voxels with a WM signal intensity > 0.99. The thresholding procedures of the MD and FA images were performed with these procedures with the template after careful normalization of each individual's images and not with the individual images' signal intensities. We then applied this mask image to the normalized FA image; therefore, we retained only areas that are highly likely to be white matter from the normalized FA images. These images were smoothed (6 mm full-width half-maximum) and carried through to the second-level analyses of FA.

A smaller smoothing value was chosen in FA because we believe FA values differ substantially among adjacent tracts, making larger smoothing values unfavorable.

These preprocessing procedures utilized the new SPM8 segmentation and modified DARTEL procedures that take into account the FA signal distribution in white matter tissues and that effectively solve the misalignment problem of voxel-based analyses of FA [10]. By applying the masks of tissue probability >0.99 before smoothing, this method also effectively alleviates the partial volume effects and the problems of signal contamination from other tissues in voxel-based analyses of FA [10]. For the quantitative and visual demonstration of the validity of these methods, see supplemental online material for our previous work [9].

We did not use T1 weighted structural images for these preprocessing procedures. As described previously "*This is because T1 weighted structural images and EPI images have apparent differences due to the distortion caused by 3T MRI and simply it is apparently not suited for the accurate and precise segmentation and normalization images of MD maps.*" [11].

Supplemental Table 1. Correlation coefficients and permutation based p-values for the associations between the spherical equivalent, significant correlated parameters, and total volume measurements.

	1	2	3	4	5	6	7	8	9	10
1 spherical equivalent	-									
2 total intracranial volume	−0.066, $<2 \times 10^{-16}$	-								
3 total gray matter volume	−0.009, 1	0.743, $<2 \times 10^{-16}$	-							
4 total white matter volume	−0.024, 0.092	0.824, $<2 \times 10^{-16}$	0.702, $<2 \times 10^{-16}$	-						
5 total CSF volume	−0.099, $<2 \times 10^{-16}$	0.791, $<2 \times 10^{-16}$	−0.006, $<2 \times 10^{-16}$	0.156, $<2 \times 10^{-16}$	-					
6 MD in the splenium	−0.123,	0.181,	0.042,	0.051,	0.191,	-				

	$<2 \times 10^{-16}$	$<2 \times 10^{-16}$	0.008	$<2 \times 10^{-16}$	$<2 \times 10^{-16}$					
7 MD in the left temporal area	−0.094,	−0.078,	−0.101,	−0.107,	0.017,	0.079,	-			
	$<2 \times 10^{-16}$	0.594	0.012	0.263	0.023	$<2 \times 10^{-16}$				
8 preoccupation	−0.085,	−0.052,	−0.066,	−0.032	-0.011,	0.049,	-0.031	-		
	$<2 \times 10^{-16}$	1	0.522	0.243	1	0.241	0.091			
9 Paranoia Checklist -	−0.092,	0.047,	−0.004,	−0.016,	0.073,	0.029,	0.005,	0.005,	-	
Conviction	$<2 \times 10^{-16}$	0.086	1	1	0.005	0.249	1	0.882		
10 study time	−0.092,	−0.081,	−0.036,	−0.019.	0.030,	0.014,	0.035,	0.037,	0.009,	—
	$<2 \times 10^{-16}$	1	0.203	0.403	1	1	0.151	0.119	0.980	
11 monthly exercise	0.061	0.068	0.053	0.016	0.074	0.035	-0.041	0.034	-0.032	-0.006
	4×10^{-4}	0.012	0.039	0.312	6×10^{-4}	0.070	0.151	0.160	0.548	1

References

1. Takeuchi, H. et al. Degree centrality and fractional amplitude of low-frequency oscillations associated with Stroop interference. *Neuroimage* **119**, 197-209 (2015).
2. Takeuchi, H. et al. Effects of Multitasking-Training on Gray Matter Structure and Resting State Neural Mechanisms. *Hum. Brain Mapp.* **35**, 3646-3660 (2014).
3. Oldfield, R.C. The assessment and analysis of handedness: the Edinburgh inventory. *Neuropsychologia* **9**, 97-113 (1971).
4. Takeuchi, H. et al. Global associations between regional gray matter volume and diverse complex cognitive functions: evidence from a large sample study. *Scientific Reports* **7**, article 10014 (2017).
5. Takeuchi, H. et al. The structure of the amygdala associates with human sexual permissiveness: Evidence from voxel-based morphometry. *Hum. Brain Mapp.* **36**, 440-448 (2015).
6. Ashburner, J. & Friston, K.J. Voxel-based morphometry-the methods. *Neuroimage* **11**, 805-821 (2000).
7. Takeuchi, H. et al. Impact of videogame play on the brain's microstructural properties: Cross-sectional and longitudinal analyses. *Mol. Psychiatry* **21**, 1781-1789 (2016).
8. Takeuchi, H. et al. White matter structures associated with empathizing and systemizing in young adults. *Neuroimage* **77**, 222-236 (2013).
9. Takeuchi, H. et al. White matter structures associated with empathizing and systemizing in young adults. *Neuroimage* **77**, 222-236 (2013).
10. Smith, S.M. et al. Tract-based spatial statistics: voxelwise analysis of multi-subject diffusion data. *Neuroimage* **31**, 1487-1505 (2006).

11. Takeuchi, H. et al. Mean diffusivity of globus pallidus associated with verbal creativity measured by divergent thinking and creativity-related temperaments in young healthy adults. *Hum. Brain Mapp.* **36**, 1808-1827 (2015).
